# Genetic assortative mating for schizophrenia and bipolar disorder

**DOI:** 10.1192/j.eurpsy.2022.2304

**Published:** 2022-08-23

**Authors:** Oskar Hougaard Jefsen, Ron Nudel, Yunpeng Wang, Jonas Bybjerg-Grauholm, Nicoline Hemager, Camilla A. J. Christiani, Birgitte K. Burton, Katrine S. Spang, Ditte Ellersgaard, Ditte L. Gantriis, Kerstin Jessica Plessen, Jens Richardt M. Jepsen, Anne A. E. Thorup, Thomas Werge, Merete Nordentoft, Ole Mors, Aja Neergaard Greve

**Affiliations:** 1 Psychosis Research Unit, Aarhus University Hospital, Central Denmark Region, Aarhus, Denmark; 2 iPSYCH, The Lundbeck Foundation Initiative for Integrative Psychiatric Research, Aarhus, Denmark; 3 CORE – Copenhagen Research Centre for Mental Health, Mental Health Centre Copenhagen, Copenhagen University Hospital, Copenhagen, Denmark; 4 Centre for Lifespan Changes in Brain and Cognition, Department of Psychology, University of Oslo, Oslo, Norway; 5 Center for Neonatal Screening, Department for Congenital Disorders, Statens Serum Institute, Copenhagen, Denmark; 6 Child and Adolescent Mental Health Centre – Research Unit, Mental Health Services in the Capital Region of Denmark, Copenhagen, Denmark; 7 Department of Clinical Medicine, Faculty of Health and Medical Sciences, University of Copenhagen, Copenhagen, Denmark; 8 Division of Child and Adolescent Psychiatry, Department of Psychiatry, University Hospital Lausanne and University of Lausanne, Lausanne, Switzerland; 9 Mental Health Services in the Capital Region of Denmark, Center for Neuropsychiatric Schizophrenia Research and Center for Clinical Intervention and Neuropsychiatric Schizophrenia Research, Hellerup, Denmark; 10 Institute of Biological Psychiatry, Mental Health Centre Sct. Hans, Mental Health Services Copenhagen, Roskilde, Denmark

**Keywords:** Assortative mating, bipolar disorder, educational attainment, polygenic scores, schizophrenia

## Abstract

**Background:**

Psychiatric disorders are highly polygenic and show patterns of partner resemblance. Partner resemblance has direct population-level genetic implications if it is caused by assortative mating, but not if it is caused by convergence or social homogamy. Using genetics may help distinguish these different mechanisms. Here, we investigated whether partner resemblance for schizophrenia and bipolar disorder is influenced by assortative mating using polygenic risk scores (PRSs).

**Methods:**

PRSs from The Danish High-Risk and Resilience Study—VIA 7 were compared between parents in three subsamples: population-based control parent pairs (*N*=198), parent pairs where at least one parent had schizophrenia (*N*=193), and parent pairs where at least one parent had bipolar disorder (*N*=115).

**Results:**

The PRS for schizophrenia was predictive of schizophrenia in the full sample and showed a significant correlation between parent pairs (*r*=0.121, *p*=0.0440), indicative of assortative mating. The PRS for bipolar disorder was also correlated between parent pairs (*r*=0.162, *p*=0.0067), but it was not predictive of bipolar disorder in the full sample, limiting the interpretation.

**Conclusions:**

Our study provides genetic evidence for assortative mating for schizophrenia, with important implications for our understanding of the genetics of schizophrenia.

## Introduction

Partner resemblance occurs for a wide range of human traits and may be caused by a variety of mechanisms. Partner resemblance may be caused by *assortative mating*, whereby people with similar traits mate more frequently than would be expected by chance alone [1], *convergence* [2, 3], whereby partners become more similar as they live together, and *social homogamy* [4], whereby individuals are more likely to mate with those living in a similar environment (leading to their being influenced by similar exposures). Importantly, these mechanisms of partner resemblance have different consequences for the population from the genetic perspective. When assortative mating operates for a heritable trait in a given population, the genotypic and phenotypic variances for that trait increase in that population [5]. For example, tall women are more likely to select tall men [6], and, over generations, this tendency increases the population-level variance in height until an equilibrium is reached. Similarly, the prevalence of a heritable disorder may increase when assortative mating for that disorder and/or assortative mating for a genetically correlated trait, that is, *secondary assortment* [7], operate. In contrast, *convergence* and *social homogamy* have no direct genetic consequences [5, 8]. Assortative mating is well-described for many traits including intelligence [9, 10], educational attainment [11–13], and height [14, 15], as described above.

Partner resemblance is observed for many psychiatric disorders [10, 16, 17], and this could, in principle, be due to assortative mating, convergence, and/or social homogamy, as outlined above. Knowing the cause of partner resemblance for psychiatric disorders is important for understanding its population-level genetic consequences. One way to disentangle assortative mating from other mechanisms of partner resemblance is to use an instrumental variable associated with the trait of interest (psychiatric illness). This trait must not be directly observed by potential mates; it must be fixed in the context of a given individual (and therefore resistant to *convergence*), and it must be independent of social homogamy. Polygenic risk scores (PRSs) derived from genome-wide association studies (GWAS), which represent an individual’s (additive) genetic predisposition to having a trait or a disease, may be used as such instrumental variables. Accordingly, a positive correlation between partners’ PRS for a trait would provide evidence for trait-specific genetic assortative mating with consequences for population-level genetics. Trait-specific genetic assortative mating has previously been demonstrated for educational attainment, height, body mass index, smoking, and alcohol consumption [11–13, 15, 18–21], but it is not commonly investigated in the context of psychiatric disorders. Yengo et al. [13] investigated genetic assortative mating for 32 complex traits and found statistically significant correlations between partners’ PRSs for height and educational attainment, but not for any psychiatric disorder, after correction for multiple comparisons.

*The Danish High Risk and Resilience Study—VIA 7* [22] provides a unique opportunity for investigating genetic assortative mating for schizophrenia and bipolar disorder, two severe psychiatric disorders with high heritabilities in the Danish population [23]. The *VIA 7* study is a population-based cohort study of 522 children born to a parent with schizophrenia or bipolar disorder, or to parents with neither of those disorders, with extensive clinically obtained bio-psycho-social (including genetic) information on parents and children. We recently reported evidence for phenotypic partner resemblance for psychiatric disorders (i.e., schizophrenia, bipolar disorder, major depressive disorder, and others), social functioning, intelligence, working memory, and processing speed in this cohort [24]. Here we focus on trait-specific genetic assortative mating for schizophrenia and bipolar disorder. This enabled us to disentangle potential mechanisms underlying the observed partner resemblance for schizophrenia and bipolar disorder. Furthermore, by investigating genetic liability to schizophrenia and bipolar disorder instead of the disorders *per se*, we were able to capture the effects of *secondary assortment* [7]. To do this, we correlated PRSs between partners for four traits:

1. Educational attainment—a “positive control” (given its well-established heritability, assortative mating patterns, and prior evidence for genetic assortative mating [11–13, 25]).
2. Bone mineral density—a “negative control” (given its high heritability, but no genetic or phenotypic assortative mating [12, 26]).
3. Schizophrenia.
4. Bipolar disorder.

We hypothesized that partners’ PRSs would be significantly correlated for schizophrenia and bipolar disorder.

## Methods

### Sample

A population-based cohort study of 522 7-year-old children at familial high risk of schizophrenia or bipolar disorder (or age-matched controls) was established in 2013, *The Danish High Risk and Resilience Study—VIA 7* (Hereafter referred to as *VIA 7*). The cohort was randomly drawn from a sample of children in the Danish Civil Registration System born between September 1, 2004, and August 31, 2009 and consisted of three groups: (a) 202 children with at least one parent diagnosed with schizophrenia spectrum psychosis (familial high-risk of schizophrenia, FHR-SZ), (b) 120 children with at least one parent diagnosed with bipolar disorder (familial high-risk of bipolar disorder, FHR-BP), and (c) 200 children of parents never diagnosed with schizophrenia or bipolar disorder in the Danish Psychiatric Central Research Register (population-based controls, PBCs). It is also important to note that selection into the FHR groups only depended on one parent—the “index” parent—whereas there was no ascertainment for the non-index parent. Accordingly, a high proportion of cases of schizophrenia or bipolar disorder (or higher PRSs) among non-index parents would be either due to chance, or due to mechanisms driving schizophrenia/bipolar disorder cases together in pairs, such as assortative mating. Furthermore, as the recruitment was centered on the children, families were not more likely to be in the sample if two parents had schizophrenia/bipolar disorder, compared to only one. Other psychiatric diagnoses (than schizophrenia or bipolar disorder) in the parents were not considered reasons for exclusion from the PBC sample. The FHR-SZ and PBC groups were matched on urbanicity, community, gender, and exact age (within a 3-month interval) of the child. A minority of the children had siblings also included in the cohort, and so the number of parent pairs was smaller than the number of children.

### Genotype data and quality control

DNA samples from a subset of the *VIA 7* study participants (children and biological parents) who contributed a sample were genotyped on the Illumina PsychChip v1-1_15073391_C array and underwent extensive quality control (QC), as described in our previous studies [27, 28]. We briefly repeat the QC steps here: initial QC on raw genetic data: individuals with low call rates or discordant sex information were removed in the first step, as were markers with a Gentrain score<0.3. Further QC with PLINK v.1.90b5.2 [29, 30] included the following: individuals and markers with Mendelian error rates exceeding 1% were removed. Genotypes with remaining Mendelian errors below this threshold were set to missing. Markers with >5% missing data were removed (at this stage all remaining individuals had <5% missing data). Individuals with extreme heterozygosity rates (with a threshold of ±3standard deviation [SD] from the sample mean) were removed. Genetic ancestry was estimated in a principal component analysis (PCA), whereby the threshold for the exclusion of samples was 2SD above or below the *VIA 7* study sample mean for either principal component (PC) 1 or PC2, using continental HapMap reference populations (CEU, CHB, JPT, and YRI) and the *VIA 7* study samples to create the PC space, as detailed in a conventional GWAS QC protocol [31]. Individuals of divergent ancestry were removed along with their relatives, whereas the rest of the sample clustered with the CEU individuals (Europeans). Individuals who exhibited cryptic relatedness or who were less related to family members than expected from pedigree information were removed (the Pi-hat threshold for the exclusion of individuals expected to be unrelated was 0.185). A Hardy–Weinberg Equilibrium (HWE) *p*-value exclusion threshold of 1×10^−6^ (irrespective of phenotype) was employed, and markers significantly deviating from HWE (i.e., failing the HWE test as per the above threshold) were removed. An inclusion minor allele frequency (MAF) threshold of 1% in founders was also employed to include only common variants. We removed one marker per pair in case of pairs of markers with identical positions included in the PsychChip. Lastly, non-autosomal markers were also removed from the dataset, resulting in a final count of 299,604 markers and 1,094 individuals. The genome build for this dataset was Human Genome version 19 (hg19).

### Pre-imputation QC, imputation and post-imputation QC

Prior to the imputation, we performed QC of the final dataset described above using the QC script “HRC or 1000G Imputation preparation and checking” v.4.2.9 downloaded from: http://www.well.ox.ac.uk/~wrayner/tools. Using this script and the reference dataset “HRC reference v1.1” for build GRCh37 (corresponding to hg19) downloaded from: ftp://ngs.sanger.ac.uk/production/hrc/HRC.r1-1, the genotype dataset was prepared for imputation. The resulting dataset was converted to variant call format (VCF) with PLINK and uploaded to the Michigan Imputation Server [32]. Both phasing and imputation were performed on the Michigan Imputation Server. The following parameters were employed: Reference Panel: HRC r1.1 2016; Phasing: Eagle V2.4 (phased output); Population: EUR; Algorithm: Genotype Imputation (Minimac4) 1.5.7; Mode: QC and imputation. Following the imputation, hard call “best guess” genotypes were kept for genotypes with probabilities of at least 0.9. Markers were removed if: they had rsq<0.3; there were other markers with the same position as theirs; they were multiallelic; they were indels; they had Mendelian error rates exceeding 1% (genotypes with remaining Mendelian errors below this threshold were set to missing); they had MAF<1% (in founders); they had a missingness rate>5%. The HWE *p*-value exclusion threshold was 1×10^−6^ (irrespective of phenotype), as before. Where possible, the imputation marker ID was changed to the rsID, based on the chromosome number and physical position using the aforementioned Haplotype Reference Consortium (HRC) reference.

### Summary statistics for the PRSs

We obtained the summary statistics from published papers or from the Psychiatric Genomics Consortium (PGC) [33, 34]. For educational attainment, the summary statistics are from the study by Lee et al. [25], which included 766,345 participants (note: the summary statistics were made available only for an analysis that had used a subset of the sample from that study). We obtained summary statistics for bone mineral density from the study by Kemp et al., which included 142,487 participants [26]. Summary statistics for schizophrenia and bipolar disorder were obtained from recent, internal meta-analyses of PGC studies from which Danish individuals had been removed. The summary statistics used in this study included 79,641 participants (34,129 cases) in the schizophrenia GWAS and 51,710 participants (20,352 cases) in the bipolar disorder GWAS discovery sample.

### Generation of PRSs

We generated PRSs with PRSice v2.2.3 [35]. We used the following parameters: clumping window of 250kb and *r* ^2^ of 0.1; scoring method: score sum; otherwise, the default parameters were used. PRSice excludes mismatched markers (when allele codes do not match even taking into account strand flips) and ambiguous markers (A/T and G/C SNPs) by default. For educational attainment, we used a *p*-value threshold of 1, which was the most predictive as reported in the original paper [25]. For bone mineral density, the original study did not examine PRS, and, as we did not have this trait in our study, we chose to use a *p*-value threshold of 1 in this case as well, in line with previous recommendations [36, 37] and to conform to the aforementioned threshold. We used the same threshold for schizophrenia and bipolar disorder, and we tested the predictive power of the PRS for these disorders by performing logistic regressions with the *glm* function in R v3.6.3 [38] using schizophrenia or bipolar disorder as the outcome and the respective PRS (standardized across the entire sample) as a predictor, together with covariates for sex, age at inclusion and 20 PCs (as per below), in the sample of unrelated parents. We calculated Nagelkerke’s R^2^ (NR^2^) measures using the *rsq.n* function of the rsq package v1.1 for R [39], whereby the NR^2^ for each PRS was calculated as the difference between the NR^2^ of the full model (outcome regressed on PRS and covariates) and the NR^2^ of a model with only the covariates.

### Calculation of principal components and estimation of relatedness in the QCed sample

We repeated the PCA procedure (computing the first 20 principal components) within the QC-passing individuals in the *VIA 7* study sample to generate PCs for use as covariates in downstream analyses. We also repeated the pairwise identity by descent (IBD) estimation within the QC-passing individuals. Both analyses were done after pruning markers and removing markers from high LD regions, as detailed in the protocol by Anderson et al. [31] cited earlier.

### Statistical analyses

We investigated pairwise correlations between parents’ PRS for educational attainment, bone mineral density, schizophrenia, and bipolar disorder, respectively, in STATA 16 (StataCorp. 2019, *Stata Statistical Software: Release 16.* College Station, TX: StataCorp LLC). Educational attainment and bone mineral density were only investigated as positive and negative control tests, respectively. Thus, we only performed positive and negative control tests in the total sample and the PBC sample, as we had no prior evidence to support either the presence (for the positive control) or absence (for the negative control) of assortative mating for these traits within populations with schizophrenia or bipolar disorder. To test for a significant difference between the correlation estimates in the total sample and the PBC subsamples (overlapping samples), we bootstrapped the difference between the coefficients of the two samples in question (with 10,000 repetitions) and performed a two-sided *z* test for the difference between the coefficients [40], based on the original sample coefficients and the standard deviation of the bootstrap estimate distribution. The bootstrapping was performed to account for the unknown covariance in the denominator of the z statistic due to sample overlap. To adjust for potential residual population stratification that could bias the main analysis, we repeated the main analyses using PRSs residualized by the first 20 PCs.

## Results

### Study sample

DNA samples from 296 parent pairs were obtained. Of these, 279 pairs passed QC (see Table 1).

**Table 1.** Study sample.

Number of parent pairs	PBC parents	FHR-SZ parents	FHR-BP parents
Original *VIA 7* sample	198	193	115
Genotyped and passed QC			
None	34 (17%)	56 (28%)	20 (17%)
One parent	36 (18%)	58 (29%)	38 (32%)
Both parents	130 (65%)	88 (44%)	61 (52%)

*Note: The number of parent pairs in the VIA 7 study, along with the number of parents that were genotyped and passed QC, was divided into samples. Note that the FHR-SZ/BP parent pairs consist of index parents (registered with schizophrenia/bipolar disorder) and a co-parent.*

Abbreviations: FHR-BP, familial high-risk of bipolar disorder; FHR-SZ, familial high-risk of schizophrenia; PBC, population-based controls; QC, quality control.

### PRS prediction of schizophrenia and bipolar disorder phenotypes

PRS for schizophrenia was significantly predictive of schizophrenia diagnosis (PRS OR=1.46, *p*=0.0005, and NR^2^=1.2%) in the total sample of individuals (non-paired). The PRS for bipolar disorder was not significantly predictive of a diagnosis of bipolar disorder in the total sample (PRS OR=1.14, *p*=0.291, and NR^2^=0.2%).

### Correlations between parents’ PRSs

We performed correlational analyses for the PRS for educational attainment, bone mineral density, schizophrenia, and bipolar disorder between parents (see Table 2). We found evidence for genetic assortative mating for educational attainment in both the total sample (*r*=0.193, *p=*0.001) and the PBC subsample (*r*=0.224, *p=*0.010), in line with what we would expect from our positive control. We found no significant correlation between parents’ PRS for bone mineral density, again, in line with what we would expect. These results support our design and our ability to detect PRS-based assortative mating in our study sample.

**Table 2.** Correlations between parents’ PRSs.

	Total sample (*n*=279 pairs)		PBC parents (*n*=130 pairs)		FHR-SZ parents (*n*=88 pairs)		FHR-BP parents (*n*=61 pairs)	
**Control traits**	*r*	*p*	*r*	*p*	*r*	*p*	*r*	*p*
Educational attainment	0.193	0.001	0.224	0.010	NA	NA	NA	NA
Bone mineral density	−0.015	0.798	−0.058	0.509	NA	NA	NA	NA
**Tested traits**								
Schizophrenia	0.121	0.044	0.150	0.088	0.006	0.953	0.151	0.246
Bipolar disorder	0.162	0.007	0.203	0.021	0.115	0.284	0.131	0.313

*Note*: Pairwise correlations between parents’ polygenic risk scores (PRSs) for educational attainment, bipolar disorder, schizophrenia, and bone mineral density. The positive and negative controls were used only in the total sample and PBC, as we had no a priori knowledge of a potential association with these PRS in the context of psychiatric disorders (and hence they would not be true *controls* in those cases). Note that the FHR-SZ/BP parent pairs consist of index parents (registered with schizophrenia/bipolar disorder) and a co-parent.

Abbreviations: FHR-BP, familial high-risk of bipolar disorder; FHR-SZ, familial high-risk of schizophrenia; PBC, population-based controls; PRS, polygenic risk scores; NA, not applicable.

We found a nominally significant correlation between parents’ PRS for schizophrenia in the total sample (*r*=0.121, *p*=0.0440) suggestive of genetic assortative mating for schizophrenia risk. We observed a similar, albeit non-significant, correlation for the schizophrenia PRS in the PBC-subsample (*r*=0.150, *p*=0.088) and FHR-BP-subsample (*r*=0.158, 0.220), but not in the FHR-SZ-subsample (*r*=0.0064, 0.953). We bootstrapped the difference between the correlation coefficients in the total sample and the PBC subsample and found no statistically significant difference (*z*=−0.43, *p*=0.666). Using the same method, we found no statistically significant difference between the PBC subsample and the FHR-SZ subsample correlation coefficients (*z*=−1.12, *p*=0.263).

We found a significant correlation between parents’ PRSs for bipolar disorder (*r*=0.162, *p*=0.0066); however, this PRS was not predictive of bipolar disorder in unrelated parents in the present sample, obscuring the interpretation, as discussed below.

Repeating the analyses using PRSs residualized by the first 20 PCs, we found no significant differences between the new estimates and the previous ones (Supplementary Table S1), suggesting that the findings of the main analyses are not driven by population stratification. Moreover, our pairwise IBD estimation obtained a highest Pi_hat (proportion IBD) of only 0.0263 between any two parents in a pair, suggesting that the parents in our QCed sample are not very related to each other (mean Pi_hat=0.0056, SD=0.0067).

We explored between-parent correlations for schizophrenia and bipolar disorder PRSs under different *p*-value thresholds and found that the between-parent correlations were absent at the lowest *p*-value thresholds, as shown in Supplementary Figure S1.

Finally, we explored potential cross-trait correlations in parents’ PRSs, but found no clear evidence for cross-trait assortative mating (Supplementary Tables S2–S5), except for a strong correlation between the fathers’ PRS for educational attainment and mothers’ PRS for bipolar disorder (*r*=0.257, *p*=0.003).

## Discussion

We investigated genetic assortative mating for schizophrenia and bipolar disorder using PRSs. In the control analyses, we found significant genetic evidence for assortative mating for educational attainment, but not for bone mineral density, replicating previous findings for educational attainment and providing support for our study design [11–13, 19]. We found a correlation between parents’ PRS for schizophrenia, indicative of assortative mating for schizophrenia risk. We also found a significant correlation between parents’ PRS for bipolar disorder; however, the interpretation of this finding was hindered by the fact that this PRS was not predictive of bipolar disorder in our sample. This could be due to the fact that both the discovery sample and the bipolar disorder subset of the target sample were both small relative to the other discovery samples and the other groups in our study, respectively. We found no clear evidence of cross-trait assortative mating, but it should be emphasized that we only examined a small subset of the possible combinations of PRSs between parents, that is, we tested only correlations between mothers and fathers.

We previously reported significant spousal resemblance for schizophrenia, but not for bipolar disorder, based on the same sample as the one used here [24]. A similar pattern was found in the Swedish register-based study by Nordsletten et al. [17], who reported strong tetrachoric correlations for schizophrenia (0.44) and weaker correlations for bipolar disorder (0.16). The present findings, based on PRSs, indicate that the spousal resemblance in schizophrenia is driven, at least in part, by assortative mating. Our findings cannot be the result of *convergence* (as a person’s PRS does not change over time) and are unlikely to be the result of social homogamy or population stratification, for the following reasons. First, QC removed individuals of non-European ancestry and related individuals, and, in general, the Danish population shows very large population homogeneity [41]. Second, the parents included in the study showed very low relatedness. Finally, residualizing the PRS for the first 20 PCs did not affect the correlations between parents, suggesting that population stratification is not driving the observed correlation.

Assortment may, hypothetically, be mediated through correlated traits (*secondary assortment*). For example, schizophrenia and bipolar disorder are both genetically correlated with several personality traits [42, 43], and polygenic risk for schizophrenia and bipolar disorder is predictive of several socio-demographic, somatic, behavioral, and psychological traits [44]. Accordingly, the assortment may not occur for the illness per se, but rather for subclinical or entirely nonclinical traits. Here, by analyzing PRSs for the illnesses, rather than the illnesses themselves, we can capture a full continuum of liability, rather than only its extremes. Hypothetically, assortative mating could be affected differentially by illness liability and manifest illness. Indeed, we observed stronger correlations for the schizophrenia PRS in the PBC sample and in the total sample compared to that observed in the schizophrenia sample, although the difference in coefficients was not statistically significant. This difference could potentially be explained by a dependence of psychiatric trait-associated assortative mating on the clinical status of the individuals. For example, assortment on subclinical or nonclinical traits related to the disorder (e.g., personality) could be attenuated if the secondary trait is obscured or overshadowed by the manifest illness. Or an individual’s state of illness could alter their partner preferences. Alternatively, it may be explained by collider stratification bias (Supplementary Figure S2). In theory, conditioning on index-parent illness could open up a biasing path between the two parents’ PRSs (for that illness) via any unmeasured variable that (a) affects both the risk of illness and (b) affects, or shares a common cause with, the PRS in the non-index parent (see Supplementary Figure S2). Assuming that this unmeasured variable has a similar direction of effect on both the risk of illness and the PRS in the non-index parent, the biasing path would induce a negative association between the two parent’s PRSs (and could not be driving the positive association observed here). The strength of this potential bias is likely limited, but could in theory explain the attenuated correlations in the samples with illness.

### Strengths and limitations

The results of our study should be assessed in the context of several factors. One limitation of our study is the size of our sample, which included only 279 parent pairs post genetic QC and may limit the power of our study. Other studies have used much larger samples in assessing genetic assortative mating for various traits, including schizophrenia for example, a study that used the UK Biobank dataset. However, we see our study as being complementary to large-scale analyses in that it offers unique types of data that are not obtainable in large datasets such as the UK Biobank. First, the parents included in our study are by definition confirmed to be couples who have had children, whereas the study by Yengo et al. [13] ascertained partnership (and not all partners have children), and did so indirectly, with the potential for partnership misclassification. Second, for psychiatric disorders, the UK Biobank typically has relatively few cases; for example, only 572 diagnoses of ICD-10F20 codes are available as of now (https://biobank.ndph.ox.ac.uk/crystal/field.cgi?id=41202, accessed 27 January 2022), and presumably these do not include many couples with children. Thus, our study offers the possibility of comparing PRS correlations across parents who may have schizophrenia or bipolar disorder (or neither), which, as we show, may be different across these different groups, with implications for understanding the contribution of the genetic similarity (with respect to disease risk) across parents in pairs in different groups. In fact, in parents without schizophrenia or in the total sample, our results, albeit showing a larger effect, agree with those reported by Yengo et al. [13], which obtained a between-mate correlation of 0.0205 (*p*=0.006) for schizophrenia PRS. As shown in a recent study, PRS correlations themselves are biased upwards in the presence of assortative mating [45]. Although this does not challenge the qualitative interpretation of our results (the presence of assortative mating), it means that our point estimates should not be taken as direct metrics of the strength of assortative mating.

In conclusion, we find genetic evidence for assortative mating for schizophrenia, but no clear evidence for a similar effect in bipolar disorder. Our findings have implications for other lines of research in psychiatric genetics, as the presence of assortative mating for schizophrenia implies bias in the heritability estimates for schizophrenia and Mendelian randomization analyses on schizophrenia [46, 47]. Due to the small sample size of the present study, our findings should be interpreted as an intermediate step toward a stronger evidence base, and we strongly encourage replication in larger datasets. In the future, growing sample sizes in psychiatric genetics are likely to yield stronger PRS prediction and will hopefully allow more powerful investigations into assortative mating for psychiatric disorders.

## Data Availability

Access to the datasets used in this study may be granted by the VIA PIs upon reasonable request.

## Supplementary material

For supplementary material accompanying this paper visit https://doi.org/10.1192/j.eurpsy.2022.2304. click here to view supplementary material
