# Supplementary material for: Genetic assortative mating for schizophrenia and bipolar disorder
Source: Eur Psychiatry. 2022 Aug 23;65(1):e53. doi: 10.1192/j.eurpsy.2022.2304 (PMC9491077; doi:10.1192/j.eurpsy.2022.2304)
Supplement: Supplementary file 1 [file S0924933822023045sup001.docx]

**Supplementary information**

**Genetic assortative mating for schizophrenia and bipolar disorder**

**Authors**

Oskar Hougaard Jefsen^1^ †

Ron Nudel^2, 3^ †

Yunpeng Wang^4^

Jonas Bybjerg-Grauholm^2, 5^

Nicoline Hemager^2, 3, 6^

Camilla A. J. Christiani^2, 3^

Birgitte K. Burton^2, 6, 10^

Katrine S. Spang^2, 6^

Ditte Ellersgaard^2, 3^

Ditte L. Gantriis^1, 2^

Kerstin Jessica Plessen^2, 6, 7^

Jens Richardt M. Jepsen^2, 3, 6, 8^

Anne A. E. Thorup^2, 6, 10^

Thomas Werge^2, 9, 10^

Merete Nordentoft^2, 3, 10^

Ole Mors^1, 2^

Aja Neergaard Greve* ^1, 2^

† These authors contributed equally to this work

**Affiliations**

^1^ Psychosis Research Unit, Aarhus University Hospital, Central Denmark Region, Denmark.

^2^ iPSYCH, The Lundbeck Foundation Initiative for Integrative Psychiatric Research, Denmark.

^3^ CORE – Copenhagen Research Centre for Mental Health, Mental Health Centre Copenhagen, Copenhagen University Hospital, Copenhagen, Denmark

^4^ Centre for Lifespan Changes in Brain and Cognition, Department of Psychology, University of Oslo, Oslo, Norway.

^5^ Center for Neonatal Screening, Department for Congenital Disorders, Statens Serum Institut, Copenhagen, Denmark

^6^ Child and Adolescent Mental Health Centre – Research unit, Mental Health Services in the Capital Region of Denmark, Copenhagen, Denmark

^7^ Division of Child and Adolescent Psychiatry, Department of Psychiatry, University Hospital Lausanne and University of Lausanne.

^8^ Mental Health Services in the Capital Region of Denmark, Center for Neuropsychiatric Schizophrenia Research and Center for Clinical Intervention and Neuropsychiatric Schizophrenia Research

^9^ Institute of Biological Psychiatry, Mental Health Centre Sct. Hans, Mental Health Services Copenhagen, Roskilde, Denmark

^10^ Department of Clinical Medicine, Faculty of Health and Medical Sciences, University of Copenhagen, Copenhagen, Denmark

*** Corresponding author**

Aja Neergaard Greve

Psychosis Research Unit, Aarhus University Hospital

Palle Juul-Jensens Blvd. 175, 8200 Aarhus, Denmark

Email: ajagreve@rm.dk

**Supplementary Results**

|  | *PRSs* | *PRS residualized by first 20 PCs* | *Difference between correlation coefficients* | |
| --- | --- | --- | --- | --- |
| *PRS* | *Correlation coefficient, r* | | *z-score* | *p-value* |
| Educational attainment | 0.193 | 0.189 | -0.16 | 0.877 |
| Bone mineral density | -0.015 | -0.012 | 0.14 | 0.886 |
| Schizophrenia | 0.121 | 0.105 | -0.98 | 0.329 |
| Bipolar disorder | 0.162 | 0.183 | 0.97 | 0.330 |

**Supplementary Table S1.** Correlation coefficients obtained with the PRSs residualized by the first 20 principal components. These estimates are from the total sample, and two-sided z-tests. To test for a significant difference between the correlation coefficients we bootstrapped the difference between the coefficients (with 10,000 repetitions), and calculated z-scores, based on the coefficient difference and the standard deviation of the bootstrap estimate distribution.

| **Total sample** | | **Mothers’ PRS** | | | |
| --- | --- | --- | --- | --- | --- |
|  | r (p-value) | EA | BMD | SZ | BP |
| **Fathers’PRS** | EA |  | 0.056  (0.351) | 0.041  (0.497) | 0.060  (0.321) |
|  | BMD | 0.034  (0.576) |  | 0.057  (0.341) | 0.026  (0.663) |
|  | SZ | -0.014  (0.813) | 0.007  (0.912) |  | 0.007  (0.911) |
|  | BP | 0.059  (0.323) | -0.047  (0.431) | 0.087  (0.149) |  |

**Supplementary Table S2.** Cross-trait between-parent PRS correlation coefficients (and corresponding p-values) calculated in the total sample (n = 279 pairs). EA = educational attainment. BMD = bone mineral density. SZ = schizophrenia. BP = bipolar disorder.

| **Population-based controls** | | **Mothers’ PRS** | | | |
| --- | --- | --- | --- | --- | --- |
|  | r (p-value) | EA | BMD | SZ | BP |
| **Fathers’PRS** | EA |  | 0.218  (0.013) | 0.019  (0.831) | 0.257  (0.003) |
|  | BMD | 0.042  (0.635) |  | 0.110  (0.214) | 0.048  (0.587) |
|  | SZ | -0.600  (0.500) | 0.161  (0.068) |  | 0.044  (0.618) |
|  | BP | 0.079  (0.373) | -0.065  (0.465) | -0.077  (0.382) |  |

**Supplementary Table S3.** Cross-trait between-parent PRS correlation coefficients (and corresponding p-values) calculated in the sample of population-based controls (n = 130 pairs). EA = educational attainment. BMD = bone mineral density. SZ = schizophrenia. BP = bipolar disorder.

| **Familial high-risk of schizophrenia sample** | | **Mothers’ PRS** | | | |
| --- | --- | --- | --- | --- | --- |
|  | r (p-value) | EA | BMD | SZ | BP |
| **Fathers’PRS** | EA |  | -0.094  (0.382) | 0.160  (0.136) | -0.077  (0.555) |
|  | BMD | 0.074  (0.495) |  | 0.047  (0.720) | -0.009  (0.944) |
|  | SZ | -0.002  (0.983) | -0.170  (0.191) |  | 0.035  (0.792) |
|  | BP | -0.012  (0.927) | 0.103  (0.429) | 0.037  (0.777) |  |

**Supplementary Table S4.** Cross-trait between-parent PRS correlation coefficients (and corresponding p-values) calculated in the sample of familial high-risk of schizophrenia (n = 88 pairs). EA = educational attainment. BMD = bone mineral density. SZ = schizophrenia. BP = bipolar disorder.

| **Familial high-risk of bipolar disorder sample** | | **Mothers’ PRS** | | | |
| --- | --- | --- | --- | --- | --- |
|  | r (p-value) | EA | BMD | SZ | BP |
| **Fathers’PRS** | EA |  | 0.018  (0.889) | -0.069  (0.599) | -0.163  (0.129) |
|  | BMD | -0.057  (0.664) |  | -0.005  (0.966) | 0.011  (0.916) |
|  | SZ | 0.113  (0.383) | -0.136  (0.208) |  | 0.164  (0.128) |
|  | BP | 0.092  (0.392) | -0.159  (0.140) | 0.104  (0.336) |  |

**Supplementary Table S5.** Cross-trait between-parent PRS correlation coefficients (and corresponding p-values) calculated in the sample of familial high-risk of bipolar disorder (n = 61 pairs). EA = educational attainment. BMD = bone mineral density. SZ = schizophrenia. BP = bipolar disorder.


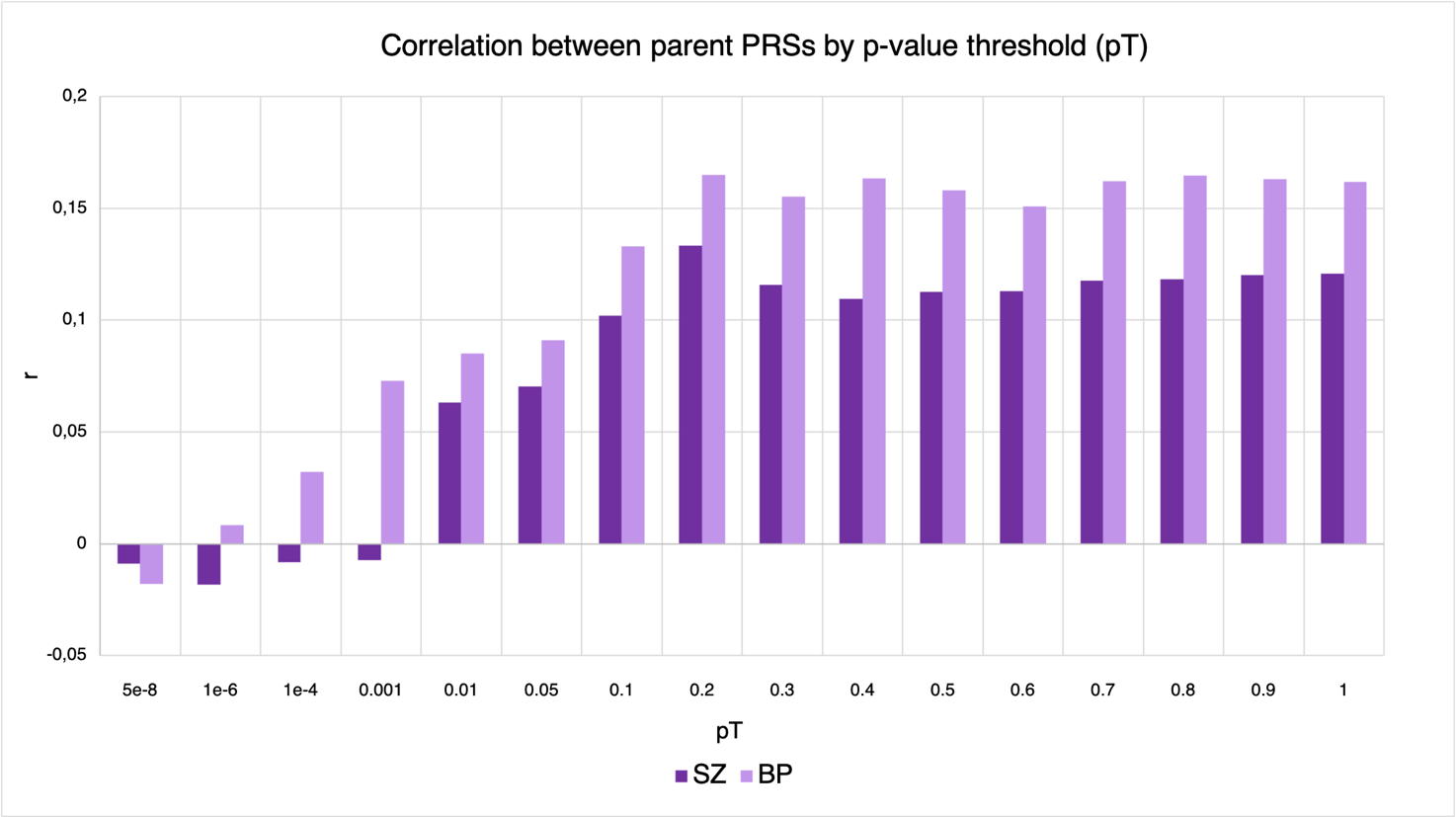


**Supplementary Figure S1.** Correlation coefficients (r) between parents’ polygenic risk scores (PRSs) for schizophrenia (SZ) and bipolar disorder (BP) using different p-value thresholds (pT) in the PRS generation.

**Supplementary Figure S2:** Directed acyclic graph (DAG) illustrating theoretical collider stratification bias in the patient samples. We are interested in the correlation between the PRS in one parent (PRS_INDEX_) and the PRS in the other parent (PRS_NONINDEX_) as a measure of genetic assortative mating. Here we assume that PRS_INDEX_ as well as other unknown variables (U_1_) both affect the risk of illness. When selecting only index-parents with an illness, we condition on illness ([Illness]). In theory, this opens up a biasing path (red arrows) through unknown variables (U_1_), given that these could cause (or share a common cause, U_2_, with) PRS in the nonindex-parent (PRS_NONINDEX_). This could in theory give rise to a negative association between PRS_INDEX_ and PRS_NONINDEX_ if the direction of effect is similar for the U_1_ → Illness path as for the U_1_ → PRS_NONINDEX_ path. The collider (illness) stratification bias described by this DAG can only generate a positive association between the two PRSs if U_1_ has opposite effects on (or association with) Illness and PRS_NONINDEX_. In other words, there would have to exist a variable (U_2_) that both increases the risk of illness and at the same time decreases the PRS of the non-index parent – a scenario that we find unlikely.
